# Moving on trial: protocol for a pilot randomised controlled trial of models of housing and support to reduce risks of COVID-19 infection and homelessness

**DOI:** 10.1186/s40814-022-00984-7

**Published:** 2022-02-01

**Authors:** Elizabeth Randell, Bethan Pell, Gwenllian Moody, Calie Dyer, Kim Smallman, Kerenza Hood, James White, Tim Aubry, Dennis Culhane, Susannah Hume, Faye Greaves, Guillermo Rodriguez-Guzman, Ligia Teixeira, Victoria Mousteri, Nick Spyropoulos, Rebecca Cannings-John, Peter Mackie

**Affiliations:** 1 Centre for Trials Research, Cardiff University, Cardiff, Wales; 2 DECIPHer, Cardiff School of Social Sciences, Cardiff University, Cardiff, Wales; 3 School of Psychology & Centre for Research, University of Ottawa, Ottawa, Canada; 4 School of Social Policy & Practice, University of Pennsylvania, Philadelphia, USA; 5 Kings College London, London, England; 6 Centre for Homelessness Impact, London, England; 7 Alma Economics, Stoke Newington Road, London, England; 8 School of Geography and Planning, Cardiff University, Cardiff, Wales

**Keywords:** Homelessness, Pilot, Randomised controlled trial, COVID-19 infection, Housing stability, Temporary accommodation

## Abstract

**Background:**

It is estimated that around 160,000 households in Britain experience homelessness each year, although no definitive statistics exist. Between March and September 2020, as part of the initial ‘Everyone In’ government response to COVID-19 in England, 10,566 people were living in emergency accommodation and nearly 18,911 people had been moved into settled accommodation. However, some forms of temporary accommodation may not be suitable as shared facilities make it impossible for people to adhere to government guidelines to reduce the spread of COVID-19.

**Methods:**

This is parallel group, pilot randomised controlled trial. The target is to recruit three local authorities, each of which will recruit 50 participants (thus a total of approximately 150 participants). Individuals are eligible if they are aged 18 and over, in a single-person homeless household, temporarily accommodated by the LA with recourse to public funds. Participants will be randomised to receive settled accommodation (intervention group) or temporary accommodation (control group). The intervention group includes settled housing such as Private Rented Sector (low and medium support), Social Housing (low and medium support), and Housing First (High support). The control group will maintain treatment as usual. The follow-up period will last 6 months. The primary outcome is to assess the feasibility of recruitment, retention, and acceptability of trial processes against progression criteria laid out in a traffic light system (green: all criteria are met, the trial should progress as designed in this pilot; amber: the majority of criteria are met and with adaptations to methods all criteria could be met; red: the minority of criteria are met and the pilot RCT should not proceed). Secondary outcomes include assessment of completeness of data collection at 3 and 6 months and percentage of participants consenting to data linkage, as well as a process evaluation and economic evaluation.

**Discussion:**

This trial will address feasibility questions associated with progression to a fully powered effectiveness trial of models of housing to reduce risk of COVID-19 infection and homelessness.

**Trial registration:**

ISRCTN69564614. Registered on December 16, 2020.

**Supplementary Information:**

The online version contains supplementary material available at 10.1186/s40814-022-00984-7.

## Background

No definitive statistics exist, but it is estimated that around 160,000 households in Britain experience homelessness each year [1]. The onset of the COVID-19 pandemic elicited an historic swift and determined effort to ensure people were safely accommodated, constituting a significant departure from usual practice, particularly in England [2, 3]. The direction handed down from Ministers to local authorities in England at the outset (March 2020) was unambiguous that everyone should be accommodated and that provision should be single room wherever possible, and allow for social distancing in all cases [4]. The intention was to ensure everyone had space to self-isolate and to reduce the risk of transmission of COVID-19.

Whilst the major challenge was sourcing additional emergency accommodation, some existing temporary accommodation also had to be decommissioned or adapted. Changes introduced in this provision during the pandemic included shared rooms becoming single occupancy rooms, whilst some shelters were closed [2]. Local authorities and their partners in national government, the third sector, Registered Social Landlords (RSL), and the private sector took swift action to commission a very wide range of new temporary accommodation, including hotels, B&Bs, holiday lets, university accommodation, and RSL properties. In England, between March and September 2020, as part of this initial ‘Everyone In’ government response to COVID-19, 10,566 people were living in emergency accommodation and nearly 18,911 people had been moved on to settled accommodation [5].

There is a government commitment to prevent people from going back to the streets including, potentially, through the re-opening of shelter-type accommodation [4]. Such accommodation, including shared facilities, might make it impossible for people to comply with government social distancing advice. Moreover, new homelessness applications continue to be made to local authorities and the limited supply of settled accommodation means swift access to settled accommodation will not be possible for all households.

There is a danger that financial and operational pressures on local authorities (LAs) may lead to those housed during the pandemic, having to return to shelters and hostels and given shortages in supply of settled accommodation, stays in temporary accommodation are likely to be protracted for many people. This could make future outbreaks of COVID-19 potentially more likely, as well as failing to make an impact on the broader social and economic consequences of people experiencing homelessness.

LAs have a limited provision of settled accommodation, so assistance for people experiencing homelessness usually includes a long stay in temporary accommodation. That many homeless people are currently waiting to be housed means they can be randomly allocated to different housing solutions at scale quickly. The insights drawn from the short-term impacts of permanent housing can be used to inform other local authorities’ responses to the challenges of COVID-19 and the cost-effectiveness of accommodation alternatives more broadly. Our established links with the UK government and the devolved administrations means these results can be disseminated at pace and at scale.

This protocol outlines a unique time limited opportunity to conduct the first ever randomised controlled trial in the UK, to evaluate the feasibility and acceptability of randomising participants to Settled Accommodation (SA) or Temporary Accommodation (TA) with the aim of preventing COVID-19 infection and reducing housing instability for people experiencing homelessness in England. Including an embedded process and economic evaluation.

## Methods

### Objectives

The primary objectives are to assess the following:

1. The feasibility of recruiting local authorities and eligible participants.
2. Recruitment rates of participants and retention through 3 months and 6 months post-randomisation follow-up data collection.
3. The acceptability of the trial and its processes, including randomisation, to single homeless households and local authorities and their willingness to participate in a definitive trial.

In addition, following secondary objectives will be addressed:

1. Adherence to the trial allocation, reach and fidelity (i.e. whether SA is delivered as intended, works as hypothesised, is scalable and sustainable).
2. The feasibility and acceptability of proposed outcome measures for a definitive trial, including resource use and health-related quality of life data, as methods to measure effectiveness of the intervention and to conduct an embedded health economic evaluation within a definitive RCT.
3. The feasibility and acceptability of linkage to routinely collected data within a definitive RCT by assessing whether (a) participants are willing to consent for their data to be linked and (b) personal identifiers can be linked to NHS Digital routine datasets.

### Trial design

**Fig. 1:**
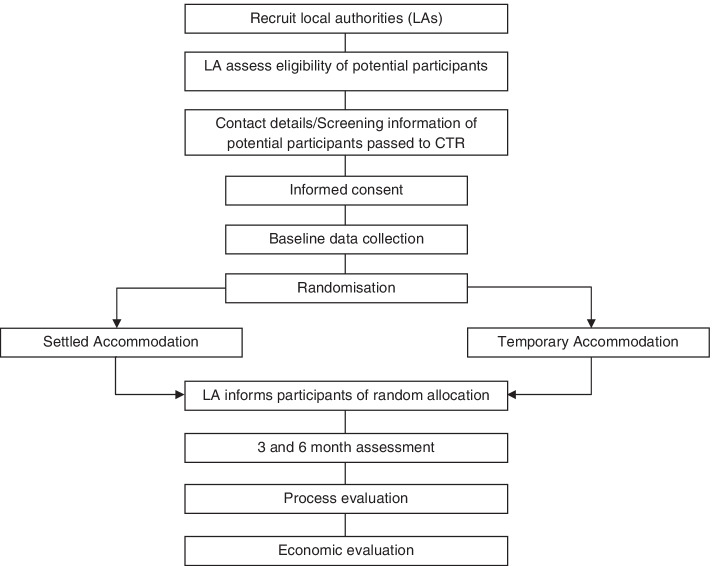
Participant timeline

This is a pilot randomised controlled trial, with embedded process and health economic evaluation. Single people experiencing homelessness, who have been temporarily accommodated by LAs in England will be randomised to receive Settled Accommodation or the comparator, Temporary Accommodation, provided by LAs. Both types of accommodation will vary by LA (Fig. 1)

.

### Trial setting

The trial target is to recruit three local authorities in England, including London/Metropolitan Boroughs and District/unitary authorities, each recruiting 50 participants (thus a total of approximately 150 participants). The trial population will be representative of all single person households who have approached local authorities for homelessness assistance. Set-up involves coordination with local authority nominated staff, a presentation about trial aims and methods, fulfilling requirements from the site (e.g. the signing of an agreement between the LA and Cardiff University) and resolving queries. Relevant site staff are trained in their roles and responsibilities including passing on trial information to clients, eligibility criteria, and transferring data securely to Cardiff University. A list of LAs taking part in the trial can be found at the ISRCTN repository (ISRCTN69564614).

### Eligibility criteria

Participants in the trial must be adults aged 18 and over, in a single person homeless household, temporarily accommodated by the LA. They will have recourse to public funds and be able to provide adequate informed consent to research participation (including competence in English at conversational level or higher).

Participants must not be in a multi-person homeless household, have no recourse to public funds, and will not be lacking in capacity to consent.

### Intervention

Participants will be randomised to either settled or temporary accommodation. Local authorities will use their standard provision of temporary accommodation (e.g. hostels) and will make offers of settled housing with the relevant level of support as they judge appropriate.

Settled housing offered can include private rented sector (PRS) (low and medium support), social housing (low and medium support), and housing first (high support). High support is provided daily, medium support is once per week, and low support is less than once a week. LAs will administer the type of settled accommodation and level of support, in relation to their own assessments.

The trial team will monitor adherence to housing allocation at the follow-up contact via questionnaires. Additionally, reasons for delays in accessing allocated accommodation and any movement between accommodation (e.g. from temporary accommodation to settled accommodation) from LAs and follow-up questionnaires will be recorded. LAs will receive trial training, where the principles and process of randomisation will be explained. The importance of providing an evidence base on the outcomes associated with settled vs temporary accommodation will also be discussed. LAs will adhere to data management and storage principles (i.e. General Data Protection Regulation, 2016).

### Outcomes

Primary outcomes are based on progression criteria:

1. Participant recruitment: Percentage of participants approached by LAs, and who are eligible, consent to take part (and thus are willing to be randomised)
2. LA recruitment: Number of LAs who entered pilot RCT as a proportion of those approached and who showed some interest.
3. Participant retention: Percentage of participants retained at final follow-up timepoint as a proportion of those recruited.
4. Adherence: LAs adhere to assignment of participant to randomised allocation.

Secondary outcomes:

1. Completeness of data collection at 3 and 6-month follow-up:
  COVID-19 infection.
  General health as measured by the EuroQol-5 Dimension (EQ-5D) [6].
  Mental health as measured by the Generalised Anxiety Disorder assessment (GAD-7) [7] and four measures from the Office for National Statistics (ONS-4) [8]. The ONS-4 captures three types of well-being: evaluative, eudemonic, and affective experience.
  Employment status.
  Income.
  Drug and alcohol use using the AUDIT-C [9].
  Service access use for mental health.
  Drug and alcohol rehabilitation/service use.
  Healthcare service use.
  Cost-effectiveness (including healthcare and mental health service use, and offending).
  Housing stability.
2. Data linkage: Percentage of participants consenting to data linkage.

### Sample size

As this is a pilot RCT to evaluate feasibility and acceptability, we will not undertake a power analysis for sample size. Based on LAs existing caseloads, we anticipate recruiting 50 participants per LA. Therefore, if we successfully recruit three LAs, we anticipate recruiting in the region of 150 participants over a 5-month recruitment period. This pilot RCT will determine response and follow-up rate estimates of effect sizes to inform a power calculation for a definitive trial if warranted given the progression criteria.

### Recruitment

LAs will have eligible participants waiting to be moved as part of the Ministry of Housing, Communities & Local Government (MHLCG) funding for move-on accommodation (Next Steps Accommodation Programme). Eligible participants will be asked for their permission to be contacted by Cardiff University staff. A list of potentially eligible participants, along with their contact details, will be passed securely onto the Centre for Trials Research, who will facilitate informed consent and questionnaire completion at each time-point, over the telephone. In addition, for those who decline contact, an anonymous participant identifier will be passed securely onto the Centre for Trials Research with no other personal identifiers.

Informed consent will be obtained before data collection takes place. This process will be facilitated by a specially trained member of the trial team, situated within Cardiff University and on the delegation log. Only those eligible potential participants, who provide verbal consent to contact, will be contacted for participation in the trial. Significant time will be allocated to explaining the trial, as well as informing potential participants of their rights pertaining to voluntary participation, confidentiality, and anonymity. Potential participants will provide verbal consent to each statement on the consent form. The researcher will only sign off verbal informed consent, if they are satisfied that the potential participant has capacity, understands and agrees to their role within the trial. Participants will then be allocated a unique number (participant identifier). Researchers will re-consent participants utilising an abridged version of the consent process at each follow-up time-point. Informed consent will be obtained separately for the qualitative component of the trial. Participants will be asked to consent to the sharing of their personal data to facilitate data linkage to routinely collected data (e.g. name, date of birth, postcode of last fixed abode). Participants will also be asked for consent to share their anonymised data with researchers within and outside of the UK and to feedback aggregated data to LAs.

### Randomisation

Allocation will occur after informed consent and completion of the baseline questionnaire using Qualtrics. The randomisation programme will be prepared in advance for each LA by the Moving On statistician and results communicated to the participating LAs, ensuring a timely allocation of housing to applicants. Randomisation will be on a 1:1 ratio and stratified (by LA) and constrained by the number of settled accommodation available in each block. Two separate lists for each LA will be constructed; one for individuals consenting to the trial and another for those that decline. Participants will be enrolled into the trial by staff trained in taking consent. Participants will be allocated to receive either the intervention or control and will be informed of their allocation by LA staff.

All parties will be blind to allocation during baseline data collection. It is not possible for Moving On trial participants, trial managers, the intervention delivery team (LA staff), or researchers involved in the process evaluation to be blind to intervention status. However, researchers at outcome data collections will attempt to remain blind to intervention status as will the statistician analysing the primary and secondary outcome data and the health economists undertaking the economic analysis. We will record any instances where allocation becomes apparent to data collectors during their interactions with participants. Potential risks of the intervention to participants are minimal but, in the case where unblinding is necessary, the allocation schedule will be available to researchers either electronically or via the independent Centre for Trials Research (CTR) at Cardiff University.

### Data collection

The trial will explore past and current experiences of people experiencing homelessness in relation to a range of life domains. The research team has sought to markedly reduce the number of questions posed and to explore the appropriateness of these questions with someone with lived experience of homelessness. However, personal questions about life experiences are a key part of the questionnaire.

We will make every effort to ensure retention. At enrolment, participants will be asked to provide alternative telephone numbers, email addresses and any other forms of communication that may be helpful to contact them. Researchers will endeavour to build a positive rapport with each participant for subsequent follow-up. Participants will also be emailed/posted vouchers as a reimbursement for their time after each questionnaire. Finally, a third-party text messaging platform (Esendex) will be used to send text messages to participants to keep in touch, or remind them of their follow-up contact.

Participants will be identified as lost to follow-up unique to a specific time point (e.g. 3 months) if it is not possible to contact them directly, via an alternative contact the participant provided (e.g. a trusted friend or family member) or via updated details from their LAs after five attempts for each contact. If they are lost to follow-up at a given time point then they will be contacted at the next follow-up time point unless they explicitly withdraw from the trial (e.g. lost at 3 months but followed up again at 6 months). This will help improve data completion rates for each time-point.

### Data management

All data will be collected using electronic data capture through a web-based survey designed specifically for this trial using Qualtrics. Data collection will be through source data provided directly from the LA or participant and entered onto the Qualtrics system database by CTR staff with appropriate restricted access.

### Progression criteria

In accordance with feasibility trial design, the Trial Management Group and independent Trial Steering Committee will assess the feasibility and acceptability of conducting a full-scale effectiveness trial using the progression criteria, based on the primary feasibility objectives of the pilot trial, described in Table 1.

**Table 1:** Table of feasibility parameters and criteria for progression to full trial

Feasibility parameter	Method of measurement	Progression criteria: green (satisfactory), amber (review), red (fail)		
		Green	Amber	Red
Participant recruitment	Percentage of participants approached by LAs, and who are eligible and consent (and thus are willing to be randomised).	≥50%	30 to <50%	<30%
LA recruitment	Number of LAs who entered trial as a proportion of those approached and who showed some interest in taking part.	≥50%	30 to <50%	<30%
Participant retention	Percentage of participants retained at final follow-up timepoint as a proportion of those recruited.	≥65%	50 to <65%	<50%
Adherence	LA adheres to assignment of participant to randomised allocation.	≥80%	50 to <80%	<50%

Although not progression criteria, fidelity of intervention delivery and adherence of participants will be measured as part of the ongoing process evaluation. Time to move to settled accommodation for participants allocated to the control arm will be examined to determine if there is a difference between the intervention and control arms.

Using the thresholds described in Table 1, we will assess progression according to a traffic light system (green: all criteria are met, the trial should progress as designed in this pilot; amber: the majority of criteria are met and with adaptations to methods all criteria could be met; red: the minority of criteria are met and the trial should not proceed).

### Statistical methods

All data will be analysed and reported in accordance with the 2016 CONSORT Extension Statement for the reporting of pilot and feasibility studies [10] and SPIRIT recommendations for reporting trials of interventions [11]. A detailed statistical analysis plan will be written prior to analysis. Statistical analysis will be performed in Stata (version 16 or higher). All analyses will be intention to treat (i.e. participants will be analysed in the groups to which they were randomised, regardless of adherence to the intervention), and missing outcome data will not be replaced (complete case analysis). As this is a feasibility trial, the purpose will be to characterise the participants and generate estimates to inform the planning of the definitive future trial guided by the primary and secondary outcomes. We will describe allocation as intended and adherence to allocation in both groups, including time to settled accommodation. We will report the difference in outcomes at 3 and 6 months between arms and report alongside 95% confidence intervals. We will report the numbers consenting to linkage and the rate of matching to NHS Digital datasets to explore whether the quality of the personal identifier data is sufficient to undertake a full-scale evaluation.

### Qualitative analysis

The trial will include an embedded process evaluation utilising a mixed-methods approach to draw together the qualitative and quantitative data. It will be conducted in accordance with the Medical Research Council’s (MRC) guidance [12]. A framework will be developed with the aim of reporting on each of the process evaluation components (Reach, Recruitment and Retention, Fidelity and Acceptability, Compliance, Contamination and Context) outlined in the MRC guidance. Data sources will be mapped against each of these components and where relevant, data will be triangulated.

We will seek to understand differences in approaches taken by the LAs, as well as implementation barriers and facilitators, sustainability and scalability of the intervention(s), and mechanism of change. The interventions are based on a Theory of Change whereby participants receiving stable accommodation will experience a reduced risk of COVID-19 infection through being more isolated and will remain stably housed. At least six people will be interviewed per LA—three service users and three staff. We will undertake a thematic content analysis of the qualitative data, in which emergent themes will be identified and organised into an analytic framework. We will explore the mechanism(s) of effect—linked to the theories of change.

### Cost-effectiveness analysis

Cost implications of different housing models and outcomes will be assessed. The Ingredients method [13] will be used, with a societal perspective and a time horizon comprising the duration of the trial. We will follow advice outlined by the Green Book [14] on economic evaluations. Economic analysis will be conducted by Alma economics and data linkage may be sought to ascertain service use between the randomised groups.

### Oversight and monitoring

A Trial Management Group (TMG) will include the chief investigators, co-applicants, collaborators, trial manager, data manager, statistician, and administrator. The TMG will meet approximately every 6 weeks throughout the course of the trial. Members will be required to sign up to the remit and conditions as set out in the TMG Charter.

The Trial Steering Committee (TSC) will meet approximately every 6 months and will comprise of an independent chairperson, two independent members and a participant representative with lived experience. Members will provide independent oversight of the trial and including matters relating to participant safety and data quality. A charter will outline the remit and conditions of the Trial Steering Committee, which members will be required to sign. Given the low-risk nature of the trial we will ask the TSC to act as Data Monitoring Committee (DMC).

### Adverse event reporting and harms

The risk of adverse events and serious adverse events relating to or attributed to the intervention are unlikely, as housing allocations are standardly provided whether or not the participant enters the trial. However, a safeguarding procedure will outline how the trial team manage safeguarding issues arising from conducting the trial questionnaires or interviews, including guidance on offering support in situ, as well as managing situations where an individual is at risk of harm. Participants will be advised that, should any serious concerns about their safety be raised, the local authority and/or police will be informed.

#### Participant safety

Where participants at risk of immediate harm, or who self-report any safeguarding concerns, the researchers will ensure participant wellbeing and appropriate services are made available. The researcher will then report this immediately (i.e. within 24 h of the event) by completing a ‘Notification of Participant Safeguarding Form’ to document the event, who will then establish best course of action based on the safeguarding concern. The form will then be reviewed and signed off by the trial Chief Investigator (or delegate) based on subsequent action that was taken.

All trial participants will receive a resource sheet with relevant services/resources in case any questions cause distress. The data manager will provide a written report of safeguarding issues during team meetings (and within 24 h of knowledge of the event) to the trial Chief Investigator (or delegate).

#### Researcher safety

Researchers will raise any concerns regarding their safety and well-being, with a member of the research team. Regular and routine supervisory meetings will monitor the impact of questionnaires and interviews on researchers’ wellbeing.

### Ethics

Significant protocol amendments and modifications will be communicated to all relevant parties in line with CTR Standard Operating Procedures and by adhering to regulatory ethical principles. Participants will be informed of the complaints procedure in the Participant Information Sheet. The Chief Investigator, local investigators, and coordinating centre do not hold insurance against claims for compensation for injury caused by participation in a trial and they cannot offer any indemnity.

### Confidentiality

Participants will be fully informed of ethical and data protection principles pertaining to confidentiality, including how their information is stored, managed, and shared in accordance with the General Data Protection Regulation 2016, and limits of confidentiality when managing safeguarding concerns.

### Dissemination

Following completion of the trial, a final report will be submitted to UKRI. We will submit our findings in high-quality, peer-reviewed journals and present results at national and international conferences. To ensure impact, we will disseminate findings to key stakeholders through the Centre for Homelessness Impact. A publication policy will be drafted and approved by the TMG. Trial findings will be fed back to participating local authorities in a debrief session and via a visual output (infographic or video) to participants.

## Discussion

This trial will address feasibility questions associated with progression to a fully powered effectiveness trial of models of housing to reduce risk of COVID-19 infection and homelessness.

This is a unique opportunity to implement a pilot randomised controlled trial design. This research will provide a pathway to development of a larger scale trial and has the potential for guiding future policy.

### Trial status

#### Protocol

This manuscript has been drafted according to Version 4.0 (May 6, 2021) of the trial protocol. The protocol has been written according to the SPIRIT (Standard Protocol Items: Recommendations for Interventional Trials) statement (Additional File 1), and the final report will follow the CONSORT (Consolidated Standards of Reporting Trials) statement.

## Supplementary Information

**Additional file 1.**

## Data Availability

The funder (UKRI) requires that all data created or repurposed during the lifetime of the grant must be made available to the UK Data Service for re-use or archiving within three months of the end of the grant.

## Abbreviations

CTR: Centre for Trials Research
DMC: Data monitoring committee
LA: Local authority
MHCLG: Ministry of Housing, Communities and Local Government
MRC: Medical research council
PRS: Private rented sector
RCT: Randomised controlled trial
RSL: Registered social landlord
SA: Settled accommodation
SPIRIT: Standard Protocol Items: Recommendations for Interventional Trials
TA: Temporary accommodation
TMG: Trial management group
TSC: Trial steering committee
